# 2-Amino-*N*-(2-benz­yloxy-3-methoxy­benzyl­idene)aniline

**DOI:** 10.1107/S1600536808017844

**Published:** 2008-06-19

**Authors:** Mohammed H. Al-Douh, Shafida A. Hamid, Hasnah Osman, Reza Kia, Hoong-Kun Fun

**Affiliations:** a School of Chemical Sciences, Universiti Sains Malaysia, 11800 USM, Penang, Malaysia; b X-ray Crystallography Unit, School of Physics, Universiti Sains Malaysia, 11800 USM, Penang, Malaysia

## Abstract

The title compound, C_21_H_20_N_2_O_2_, a Schiff base ligand, contains two independent mol­ecules (*A* and *B*) in the asymmetric unit, with similar conformations. In mol­ecule *A*, the central benzene ring forms dihedral angles of 30.79 (13) and 23.56 (13)°, respectively, with the amino and benzyl benzene rings, while in mol­ecule *B* these angles are 32.30 (13) and 13.13 (12)°. The mol­ecular structure is stabilized by intra­molecular N—H⋯N and C—H⋯O hydrogen bonds. The crystal structure is stabilized by N—H⋯N hydrogen bonds and N—H⋯π and C—H⋯π inter­actions.

## Related literature

For hydrogen-bond motifs, see: Bernstein *et al.* (1995 ▶). For bond-length data, see: Allen *et al.* (1987 ▶). For related structures, see: Al-Douh *et al.* (2006*a* ▶,*b* ▶, 2007 ▶, 2008 ▶); Corden *et al.* (1996 ▶); Govindasamy *et al.* (1999 ▶); Pozharskii *et al.* (1966 ▶).
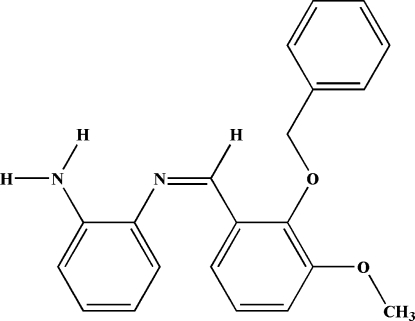


## Experimental

### 

#### Crystal data

C_21_H_20_N_2_O_2_
*M* *_r_* = 332.39
Monoclinic, 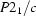

*a* = 12.0932 (2) Å
*b* = 13.7680 (3) Å
*c* = 20.5249 (4) Å
β = 99.149 (1)°
*V* = 3373.90 (11) Å^3^
*Z* = 8
Mo *K*α radiation
μ = 0.09 mm^−1^
*T* = 100.0 (1) K
0.36 × 0.18 × 0.07 mm

#### Data collection

Bruker SMART APEXII CCD area-detector diffractometer
Absorption correction: multi-scan (*SADABS*; Bruker 2005 ▶) *T*_min_ = 0.970, *T*_max_ = 0.994
37354 measured reflections
7737 independent reflections
4167 reflections with *I* > 2σ(*I*)
*R*_int_ = 0.082

#### Refinement

*R*[*F* ^2^ > 2σ(*F* ^2^)] = 0.070
*wR*(*F* ^2^) = 0.192
*S* = 1.03
7737 reflections
465 parameters
4 restraints
H atoms treated by a mixture of independent and constrained refinement
Δρ_max_ = 0.87 e Å^−3^
Δρ_min_ = −0.38 e Å^−3^

### 

Data collection: *APEX2* (Bruker, 2005 ▶); cell refinement: *APEX2*; data reduction: *SAINT* (Bruker, 2005 ▶); program(s) used to solve structure: *SHELXTL* (Sheldrick, 2008 ▶); program(s) used to refine structure: *SHELXTL*; molecular graphics: *SHELXTL*; software used to prepare material for publication: *SHELXTL* and *PLATON* (Spek, 2003 ▶).

## Supplementary Material

Crystal structure: contains datablocks global, I. DOI: 10.1107/S1600536808017844/ci2609sup1.cif

Structure factors: contains datablocks I. DOI: 10.1107/S1600536808017844/ci2609Isup2.hkl

Additional supplementary materials:  crystallographic information; 3D view; checkCIF report

## Figures and Tables

**Table 1:** Hydrogen-bond geometry (Å, °) *Cg*1, *Cg*2, *Cg*3, *Cg*4, *Cg*5 and *Cg*6 are the centroids of the C1*A*–C6*A*, C8*A*–C13*A*, C15*A*–C20*A*, C1*B*–C6*B*, C8*B*–C13*B* and C15*B*–C20*B* rings, respectively.

*D*—H⋯*A*	*D*—H	H⋯*A*	*D*⋯*A*	*D*—H⋯*A*
N2*A*—H2*AC*⋯N1*A*	0.92 (3)	2.31 (3)	2.735 (4)	108 (2)
N2*A*—H2*AC*⋯N2*B*^i^	0.92 (3)	2.49 (3)	3.229 (4)	138 (3)
N2*B*—H2*BC*⋯N1*B*	0.90 (2)	2.29 (3)	2.726 (3)	109 (2)
C7*A*—H7*A*⋯O1*A*	0.95	2.43	2.765 (3)	101
C7*B*—H7*B*⋯O1*B*	0.95	2.46	2.790 (3)	100
C14*A*—H14*A*⋯O2*A*	0.99	2.43	2.895 (3)	108
C14*B*—H14*D*⋯O2*B*	0.99	2.45	2.903 (3)	107
C21*A*—H21*C*⋯*Cg*1^ii^	0.98	2.96	3.511 (3)	117
C21*B*—H21*F*⋯*Cg*2^iii^	0.98	2.81	3.739 (3)	159
C10*A*—H10*A*⋯*Cg*3^ii^	0.95	2.60	3.500 (3)	159
C21*B*—H21*E*⋯*Cg*4^iv^	0.98	2.80	3.433 (3)	123
C21*A*—H21*B*⋯*Cg*5^v^	0.98	2.96	3.844 (4)	150
C10*B*—H10*B*⋯*Cg*6^iv^	0.95	2.66	3.587 (3)	165
N2*B*—H2*BC*⋯*Cg*6^vi^	0.90 (2)	2.83 (3)	3.288 (3)	113 (2)

Symmetry codes: (i) 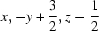; (ii) 
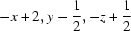
; (iii) 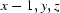; (iv) 
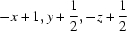
; (v) 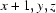; (vi) 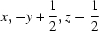.

**Fig. 1.**
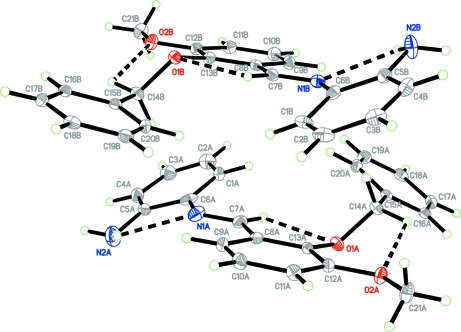
The asymmetric unit of the title compound. Displacement ellipsoids are drawn at the 30% probability level. Intramolecular interactions are shown as dashed lines.

## supplementary crystallographic information

### Comment

Mono-anil, is a Schiff base compound prepared from an equimolar amount of *o*-phenylenediamine and an aromatic or heterocyclic aldehyde as the first stage to produce 2-substituted benzimidazole derivatives following spontaneous oxidation by atmospheric oxygen (Pozharskii *et al.*, 1966). In our previous reports (Al-Douh *et al.*, 2007,2006a,b; Al-Douh *et al.*, 2008), we have reported crystal structures of 2-(2-benzyloxy -3-methoxyphenyl)-1-*H*-benzimidazole, benzyl *o*-vanillin and a derivative of the title compound, 2-amino-*N*-(2-hydroxy-3-methoxybenzylidene) benzeneamine. Continuing our investigation on the reaction mechanism of benzyl *o*-vanillin with *o*-phenylenediamine, we successfully synthesized the title compound, as a new amino benzeneamine derivative. We present here its crystal structure.

The bond lengths and angles in the title compound have normal values (Allen *et al.*, 1987) and are comparable with those a realated structure (Al-Douh *et al.*, 2008). The asymmetric unit contains two independent molecules [*A* and *B*] with almost similar conformations (Fig.1). In both A and B, the methoxy group is almost coplanar with the attached benzene ring [C21–O2–C12–C11 = -3.6 (4)° for A and -2.5 (4)° for B]. In molecule A, the C1-C6 and C15-C20 rings form dihedral angles of 30.79 (13)° and 23.56 (13)°, respectively, with the C8-C13 ring, while in B these angles are 32.30 (13)° and 13.13 (12)°. Intramolecular C—H···O and N—H···N hydrogen bonds involving O1, O2 and N1 atoms generate S(5) or S(6) ring motifs.

The crystal packing of the title compound is controlled by N—H···N hydrogen bonds, and N—H···π and C—H···π interactions (Table 1).

### Experimental

The title compound was synthesized following procedures reported earlier (Al-Douh *et al.*, 2006a,b; Al-Douh *et al.*, 2007). Single crystals suitable for *X*-ray diffraction were obtained by slow evaporation of a hexane solution at room temperature.

### Refinement

Amino H atoms were located in a difference map and their positional parameters were refined with N-H distances restrained to 0.90 (1)Å. C-bound H atoms were positioned geometrically and refined using a riding model with C-H = 0.95 Å for aromatic and methyine H, 0.99 Å for methylene H, and 0.98 Å for methyl H atoms. The *U*_iso_ values were constrained to be 1.5*U*_eq_ of the carrier atom for methyl H atoms and 1.2*U*_eq_ for the remaining H atoms. A rotating group model was used for the methyl groups. The highest peak is located at 0.60 Å from H7A and the deepest hole is located at 0.71 Å from N2A.

### Crystal data

**Table:**

C_21_H_20_N_2_O_2_	*F*_000_ = 1408
*M**_r_* = 332.39	*D*_x_ = 1.309 Mg m^−^^3^
Monoclinic, *P*2_1_/*c*	Mo *K*α radiation λ = 0.71073 Å
Hall symbol: -P 2ybc	Cell parameters from 3259 reflections
*a* = 12.0932 (2) Å	θ = 2.4–22.7º
*b* = 13.7680 (3) Å	µ = 0.09 mm^−^^1^
*c* = 20.5249 (4) Å	*T* = 100.0 (1) K
β = 99.149 (1)º	Plate, yellow
*V* = 3373.90 (11) Å^3^	0.36 × 0.18 × 0.07 mm
*Z* = 8	

### Data collection

**Table:**

Bruker SMART APEXII CCD area-detector diffractometer	7737 independent reflections
Radiation source: fine-focus sealed tube	4167 reflections with *I* > 2σ(*I*)
Monochromator: graphite	*R*_int_ = 0.082
*T* = 100.0(1) K	θ_max_ = 27.5º
φ and ω scans	θ_min_ = 1.7º
Absorption correction: multi-scan(SADABS; Bruker 2005)	*h* = −15→15
*T*_min_ = 0.970, *T*_max_ = 0.994	*k* = −17→17
37354 measured reflections	*l* = −26→26

### Refinement

**Table:**

Refinement on *F*^2^	Secondary atom site location: difference Fourier map
Least-squares matrix: full	Hydrogen site location: inferred from neighbouring sites
*R*[*F*^2^ > 2σ(*F*^2^)] = 0.070	H atoms treated by a mixture of independent and constrained refinement
*wR*(*F*^2^) = 0.192	*w* = 1/[σ^2^(*F*_o_^2^) + (0.0781*P*)^2^ + 1.4666*P*] where *P* = (*F*_o_^2^ + 2*F*_c_^2^)/3
*S* = 1.03	(Δ/σ)_max_ = 0.001
7737 reflections	Δρ_max_ = 0.87 e Å^−^^3^
465 parameters	Δρ_min_ = −0.38 e Å^−^^3^
4 restraints	Extinction correction: none
Primary atom site location: structure-invariant direct methods	

### Special details

**Table:**

Experimental. The low-temperature data was collected with the Oxford Cyrosystem Cobra low-temperature attachment.
Geometry. All esds (except the esd in the dihedral angle between two l.s. planes) are estimated using the full covariance matrix. The cell esds are taken into account individually in the estimation of esds in distances, angles and torsion angles; correlations between esds in cell parameters are only used when they are defined by crystal symmetry. An approximate (isotropic) treatment of cell esds is used for estimating esds involving l.s. planes.
Refinement. Refinement of F^2^ against ALL reflections. The weighted R-factor wR and goodness of fit S are based on F^2^, conventional R-factors R are based on F, with F set to zero for negative F^2^. The threshold expression of F^2^ > 2sigma(F^2^) is used only for calculating R-factors(gt) etc. and is not relevant to the choice of reflections for refinement. R-factors based on F^2^ are statistically about twice as large as those based on F, and R- factors based on ALL data will be even larger.

### Fractional atomic coordinates and isotropic or equivalent isotropic displacement parameters (Å^2^)

**Table:**

	*x*	*y*	*z*	*U*_iso_*/*U*_eq_	
O1A	0.99391 (15)	0.97464 (12)	0.31812 (8)	0.0232 (4)	
O2A	1.17303 (15)	0.85898 (13)	0.35794 (9)	0.0257 (5)	
N1A	0.7858 (2)	0.94192 (17)	0.14556 (13)	0.0358 (6)	
N2A	0.7063 (3)	0.9977 (2)	0.01884 (15)	0.0516 (8)	
H2AB	0.657 (2)	1.000 (3)	−0.0199 (10)	0.062*	
H2AC	0.748 (3)	0.9432 (16)	0.0317 (17)	0.062*	
C1A	0.6378 (3)	1.0482 (2)	0.18465 (15)	0.0353 (8)	
H1A	0.6636	1.0329	0.2296	0.042*	
C2A	0.5474 (3)	1.1075 (2)	0.16928 (18)	0.0401 (8)	
H2A	0.5100	1.1320	0.2032	0.048*	
C3A	0.5108 (3)	1.1317 (2)	0.10576 (17)	0.0395 (8)	
H3A	0.4500	1.1757	0.0954	0.047*	
C4A	0.5609 (3)	1.0930 (2)	0.05582 (15)	0.0331 (7)	
H4A	0.5319	1.1097	0.0115	0.040*	
C5A	0.6517 (2)	1.0309 (2)	0.06765 (14)	0.0283 (7)	
C6A	0.6950 (2)	1.0082 (2)	0.13495 (17)	0.0344 (8)	
C7A	0.8502 (2)	0.9488 (2)	0.20040 (15)	0.0311 (7)	
H7A	0.8354	0.9973	0.2308	0.037*	
C8A	0.9480 (2)	0.88351 (19)	0.21891 (13)	0.0227 (6)	
C9A	0.9735 (2)	0.8086 (2)	0.17760 (13)	0.0266 (7)	
H9A	0.9279	0.7987	0.1360	0.032*	
C10A	1.0642 (3)	0.7494 (2)	0.19684 (14)	0.0283 (7)	
H10A	1.0797	0.6980	0.1688	0.034*	
C11A	1.1335 (2)	0.76356 (19)	0.25672 (14)	0.0258 (7)	
H11A	1.1967	0.7227	0.2691	0.031*	
C12A	1.1104 (2)	0.83792 (19)	0.29868 (13)	0.0224 (6)	
C13A	1.0167 (2)	0.89782 (18)	0.27938 (13)	0.0192 (6)	
C14A	0.9679 (2)	0.94967 (19)	0.38311 (13)	0.0246 (6)	
H14A	1.0379	0.9411	0.4149	0.030*	
H14B	0.9249	0.8882	0.3808	0.030*	
C15A	0.9004 (2)	1.03068 (18)	0.40466 (12)	0.0201 (6)	
C16A	0.9483 (2)	1.09881 (19)	0.45039 (13)	0.0217 (6)	
H16A	1.0251	1.0934	0.4691	0.026*	
C17A	0.8843 (2)	1.17526 (19)	0.46915 (13)	0.0253 (7)	
H17A	0.9171	1.2208	0.5013	0.030*	
C18A	0.7739 (2)	1.1844 (2)	0.44110 (14)	0.0268 (7)	
H18A	0.7309	1.2373	0.4532	0.032*	
C19A	0.7249 (2)	1.1172 (2)	0.39544 (14)	0.0302 (7)	
H19A	0.6486	1.1238	0.3760	0.036*	
C20A	0.7880 (2)	1.0401 (2)	0.37814 (13)	0.0250 (6)	
H20A	0.7538	0.9929	0.3476	0.030*	
C21A	1.2661 (2)	0.7965 (2)	0.38025 (15)	0.0322 (7)	
H21A	1.3063	0.8206	0.4224	0.048*	
H21B	1.3167	0.7956	0.3474	0.048*	
H21C	1.2388	0.7306	0.3862	0.048*	
O1B	0.50904 (15)	0.75379 (12)	0.18231 (9)	0.0226 (4)	
O2B	0.33148 (15)	0.87429 (13)	0.14797 (9)	0.0282 (5)	
N1B	0.71887 (19)	0.77598 (16)	0.35724 (11)	0.0243 (5)	
N2B	0.7842 (2)	0.7181 (2)	0.48460 (13)	0.0408 (7)	
H2BB	0.830 (2)	0.720 (2)	0.5241 (9)	0.049*	
H2BC	0.738 (2)	0.7680 (17)	0.4697 (15)	0.049*	
C1B	0.8724 (2)	0.6776 (2)	0.32288 (15)	0.0288 (7)	
H1B	0.8507	0.6957	0.2780	0.035*	
C2B	0.9638 (3)	0.6175 (2)	0.34035 (16)	0.0328 (7)	
H2B	1.0044	0.5943	0.3075	0.039*	
C3B	0.9958 (3)	0.5914 (2)	0.40519 (16)	0.0324 (7)	
H3B	1.0582	0.5494	0.4169	0.039*	
C4B	0.9382 (2)	0.6254 (2)	0.45366 (16)	0.0313 (7)	
H4B	0.9617	0.6071	0.4983	0.038*	
C5B	0.8457 (2)	0.68672 (19)	0.43727 (14)	0.0257 (7)	
C6B	0.8112 (2)	0.71220 (18)	0.37070 (14)	0.0249 (7)	
C7B	0.6539 (2)	0.77124 (19)	0.30195 (14)	0.0234 (6)	
H7B	0.6680	0.7235	0.2709	0.028*	
C8B	0.5580 (2)	0.83669 (19)	0.28451 (13)	0.0218 (6)	
C9B	0.5342 (2)	0.9077 (2)	0.32963 (14)	0.0264 (7)	
H9B	0.5807	0.9139	0.3713	0.032*	
C10B	0.4435 (2)	0.9681 (2)	0.31328 (14)	0.0289 (7)	
H10B	0.4284	1.0164	0.3437	0.035*	
C11B	0.3737 (2)	0.95954 (19)	0.25302 (14)	0.0256 (6)	
H11B	0.3111	1.0016	0.2426	0.031*	
C12B	0.3950 (2)	0.88960 (19)	0.20775 (14)	0.0227 (6)	
C13B	0.4884 (2)	0.82822 (18)	0.22413 (13)	0.0213 (6)	
C14B	0.5337 (2)	0.78276 (19)	0.11804 (13)	0.0250 (7)	
H14C	0.5767	0.8443	0.1217	0.030*	
H14D	0.4634	0.7927	0.0868	0.030*	
C15B	0.6015 (2)	0.70273 (18)	0.09400 (13)	0.0194 (6)	
C16B	0.5587 (2)	0.64734 (18)	0.03910 (13)	0.0216 (6)	
H16B	0.4861	0.6607	0.0157	0.026*	
C17B	0.6221 (2)	0.57224 (19)	0.01840 (13)	0.0242 (6)	
H17B	0.5935	0.5355	−0.0197	0.029*	
C18B	0.7262 (2)	0.55126 (19)	0.05313 (14)	0.0251 (6)	
H18B	0.7684	0.4989	0.0396	0.030*	
C19B	0.7695 (2)	0.6061 (2)	0.10765 (14)	0.0254 (6)	
H19B	0.8417	0.5919	0.1315	0.030*	
C20B	0.7074 (2)	0.68178 (19)	0.12734 (13)	0.0227 (6)	
H20B	0.7380	0.7200	0.1644	0.027*	
C21B	0.2325 (2)	0.9329 (2)	0.13189 (15)	0.0323 (7)	
H21D	0.1931	0.9146	0.0881	0.048*	
H21E	0.2537	1.0015	0.1317	0.048*	
H21F	0.1833	0.9223	0.1648	0.048*	

### Atomic displacement parameters (Å^2^)

**Table:**

	*U*^11^	*U*^22^	*U*^33^	*U*^12^	*U*^13^	*U*^23^
O1A	0.0304 (11)	0.0202 (9)	0.0203 (10)	0.0033 (8)	0.0083 (9)	0.0007 (8)
O2A	0.0230 (11)	0.0288 (10)	0.0235 (11)	0.0054 (8)	−0.0019 (9)	−0.0040 (8)
N1A	0.0313 (15)	0.0302 (14)	0.0435 (17)	−0.0047 (12)	−0.0018 (13)	0.0055 (12)
N2A	0.045 (2)	0.071 (2)	0.0371 (18)	0.0098 (16)	0.0005 (15)	−0.0018 (17)
C1A	0.0352 (19)	0.049 (2)	0.0198 (16)	−0.0130 (16)	−0.0011 (14)	0.0015 (14)
C2A	0.041 (2)	0.0316 (17)	0.050 (2)	0.0033 (15)	0.0119 (17)	−0.0015 (16)
C3A	0.044 (2)	0.0312 (17)	0.045 (2)	−0.0025 (15)	0.0121 (17)	−0.0029 (16)
C4A	0.0277 (18)	0.0409 (18)	0.0292 (17)	−0.0002 (14)	0.0004 (14)	−0.0017 (14)
C5A	0.0225 (16)	0.0376 (17)	0.0249 (16)	−0.0042 (13)	0.0044 (13)	0.0023 (13)
C6A	0.0217 (17)	0.0251 (15)	0.055 (2)	−0.0074 (13)	0.0005 (15)	0.0126 (15)
C7A	0.0273 (17)	0.0327 (16)	0.0312 (17)	−0.0103 (13)	−0.0021 (14)	0.0136 (14)
C8A	0.0199 (15)	0.0256 (14)	0.0234 (15)	−0.0034 (12)	0.0056 (12)	0.0027 (12)
C9A	0.0276 (17)	0.0319 (16)	0.0197 (15)	−0.0084 (13)	0.0018 (13)	−0.0022 (13)
C10A	0.0361 (18)	0.0251 (15)	0.0253 (16)	−0.0009 (13)	0.0093 (14)	−0.0064 (13)
C11A	0.0270 (17)	0.0244 (14)	0.0267 (16)	0.0039 (12)	0.0061 (13)	0.0001 (12)
C12A	0.0235 (16)	0.0238 (14)	0.0197 (14)	−0.0034 (12)	0.0029 (12)	−0.0002 (12)
C13A	0.0216 (15)	0.0181 (13)	0.0188 (14)	−0.0012 (11)	0.0058 (12)	0.0011 (11)
C14A	0.0303 (17)	0.0243 (14)	0.0202 (15)	0.0018 (12)	0.0069 (12)	0.0025 (12)
C15A	0.0224 (16)	0.0228 (13)	0.0168 (14)	0.0004 (11)	0.0080 (12)	0.0044 (11)
C16A	0.0199 (15)	0.0252 (14)	0.0204 (15)	−0.0014 (12)	0.0041 (12)	0.0064 (12)
C17A	0.0361 (18)	0.0204 (14)	0.0198 (15)	−0.0012 (13)	0.0057 (13)	−0.0003 (12)
C18A	0.0299 (18)	0.0248 (15)	0.0284 (16)	0.0083 (13)	0.0127 (14)	0.0050 (13)
C19A	0.0198 (16)	0.0418 (17)	0.0297 (17)	0.0042 (13)	0.0061 (13)	0.0057 (14)
C20A	0.0218 (16)	0.0308 (15)	0.0224 (15)	−0.0021 (12)	0.0031 (12)	−0.0040 (12)
C21A	0.0276 (17)	0.0351 (16)	0.0301 (17)	0.0101 (13)	−0.0070 (14)	−0.0010 (14)
O1B	0.0265 (11)	0.0201 (9)	0.0221 (10)	0.0013 (8)	0.0070 (9)	−0.0013 (8)
O2B	0.0255 (11)	0.0281 (10)	0.0292 (11)	0.0050 (9)	−0.0010 (9)	−0.0041 (9)
N1B	0.0229 (13)	0.0244 (12)	0.0266 (14)	−0.0005 (10)	0.0068 (11)	0.0008 (10)
N2B	0.0474 (19)	0.0516 (18)	0.0213 (14)	0.0191 (14)	−0.0011 (13)	−0.0062 (13)
C1B	0.0287 (17)	0.0257 (15)	0.0348 (17)	−0.0035 (13)	0.0136 (14)	0.0047 (13)
C2B	0.0287 (18)	0.0246 (15)	0.049 (2)	0.0013 (13)	0.0168 (15)	0.0047 (14)
C3B	0.0254 (17)	0.0226 (15)	0.049 (2)	0.0021 (13)	0.0063 (15)	0.0020 (14)
C4B	0.0290 (17)	0.0251 (15)	0.0366 (18)	0.0001 (13)	−0.0045 (14)	−0.0028 (14)
C5B	0.0254 (17)	0.0235 (14)	0.0278 (16)	−0.0014 (12)	0.0030 (13)	−0.0046 (12)
C6B	0.0242 (16)	0.0185 (13)	0.0309 (17)	−0.0037 (12)	0.0013 (13)	−0.0016 (12)
C7B	0.0272 (16)	0.0232 (14)	0.0216 (15)	−0.0023 (12)	0.0093 (13)	−0.0011 (12)
C8B	0.0211 (15)	0.0233 (14)	0.0230 (15)	−0.0019 (12)	0.0092 (12)	0.0012 (12)
C9B	0.0280 (17)	0.0293 (15)	0.0223 (15)	0.0006 (13)	0.0055 (13)	−0.0036 (12)
C10B	0.0327 (18)	0.0238 (14)	0.0321 (17)	−0.0001 (13)	0.0114 (14)	−0.0079 (13)
C11B	0.0226 (16)	0.0236 (14)	0.0320 (17)	0.0036 (12)	0.0088 (13)	−0.0031 (13)
C12B	0.0221 (16)	0.0216 (14)	0.0249 (15)	−0.0008 (12)	0.0056 (12)	−0.0011 (12)
C13B	0.0232 (16)	0.0164 (13)	0.0256 (15)	−0.0015 (11)	0.0076 (12)	−0.0026 (11)
C14B	0.0290 (17)	0.0240 (14)	0.0232 (15)	0.0012 (12)	0.0074 (13)	0.0015 (12)
C15B	0.0194 (15)	0.0197 (13)	0.0196 (14)	−0.0018 (11)	0.0050 (12)	0.0032 (11)
C16B	0.0195 (15)	0.0255 (14)	0.0198 (14)	−0.0025 (12)	0.0033 (12)	0.0052 (12)
C17B	0.0292 (17)	0.0229 (14)	0.0216 (15)	−0.0069 (12)	0.0071 (13)	−0.0035 (12)
C18B	0.0268 (17)	0.0223 (14)	0.0289 (16)	0.0013 (12)	0.0131 (13)	0.0039 (12)
C19B	0.0211 (16)	0.0320 (15)	0.0239 (16)	−0.0003 (12)	0.0060 (12)	0.0049 (13)
C20B	0.0240 (16)	0.0271 (15)	0.0173 (14)	−0.0034 (12)	0.0042 (12)	−0.0024 (12)
C21B	0.0266 (17)	0.0299 (15)	0.0381 (18)	0.0077 (13)	−0.0018 (14)	0.0012 (14)

### Geometric parameters (Å, °)

**Table:**

O1A—C13A	1.377 (3)	O1B—C13B	1.385 (3)
O1A—C14A	1.459 (3)	O1B—C14B	1.454 (3)
O2A—C12A	1.358 (3)	O2B—C12B	1.357 (3)
O2A—C21A	1.432 (3)	O2B—C21B	1.437 (3)
N1A—C7A	1.266 (4)	N1B—C7B	1.275 (3)
N1A—C6A	1.417 (4)	N1B—C6B	1.413 (3)
N2A—C5A	1.363 (4)	N2B—C5B	1.383 (4)
N2A—H2AB	0.917 (10)	N2B—H2BB	0.905 (10)
N2A—H2AC	0.921 (10)	N2B—H2BC	0.905 (10)
C1A—C2A	1.361 (4)	C1B—C2B	1.381 (4)
C1A—C6A	1.431 (4)	C1B—C6B	1.403 (4)
C1A—H1A	0.95	C1B—H1B	0.95
C2A—C3A	1.350 (4)	C2B—C3B	1.374 (4)
C2A—H2A	0.95	C2B—H2B	0.95
C3A—C4A	1.378 (4)	C3B—C4B	1.384 (4)
C3A—H3A	0.95	C3B—H3B	0.95
C4A—C5A	1.383 (4)	C4B—C5B	1.399 (4)
C4A—H4A	0.95	C4B—H4B	0.95
C5A—C6A	1.432 (4)	C5B—C6B	1.408 (4)
C7A—C8A	1.486 (4)	C7B—C8B	1.467 (4)
C7A—H7A	0.95	C7B—H7B	0.95
C8A—C13A	1.393 (4)	C8B—C13B	1.388 (4)
C8A—C9A	1.401 (4)	C8B—C9B	1.408 (4)
C9A—C10A	1.373 (4)	C9B—C10B	1.374 (4)
C9A—H9A	0.95	C9B—H9B	0.95
C10A—C11A	1.387 (4)	C10B—C11B	1.387 (4)
C10A—H10A	0.95	C10B—H10B	0.95
C11A—C12A	1.394 (4)	C11B—C12B	1.391 (4)
C11A—H11A	0.95	C11B—H11B	0.95
C12A—C13A	1.407 (4)	C12B—C13B	1.407 (4)
C14A—C15A	1.491 (4)	C14B—C15B	1.503 (4)
C14A—H14A	0.99	C14B—H14C	0.99
C14A—H14B	0.99	C14B—H14D	0.99
C15A—C16A	1.387 (4)	C15B—C20B	1.383 (4)
C15A—C20A	1.387 (4)	C15B—C16B	1.391 (4)
C16A—C17A	1.396 (4)	C16B—C17B	1.393 (4)
C16A—H16A	0.95	C16B—H16B	0.95
C17A—C18A	1.373 (4)	C17B—C18B	1.374 (4)
C17A—H17A	0.95	C17B—H17B	0.95
C18A—C19A	1.382 (4)	C18B—C19B	1.383 (4)
C18A—H18A	0.95	C18B—H18B	0.95
C19A—C20A	1.387 (4)	C19B—C20B	1.381 (4)
C19A—H19A	0.95	C19B—H19B	0.95
C20A—H20A	0.95	C20B—H20B	0.95
C21A—H21A	0.98	C21B—H21D	0.98
C21A—H21B	0.98	C21B—H21E	0.98
C21A—H21C	0.98	C21B—H21F	0.98
			
C13A—O1A—C14A	116.02 (19)	C13B—O1B—C14B	116.28 (19)
C12A—O2A—C21A	117.1 (2)	C12B—O2B—C21B	116.9 (2)
C7A—N1A—C6A	116.1 (3)	C7B—N1B—C6B	119.9 (2)
C5A—N2A—H2AB	107 (2)	C5B—N2B—H2BB	109 (2)
C5A—N2A—H2AC	112 (2)	C5B—N2B—H2BC	112 (2)
H2AB—N2A—H2AC	122 (3)	H2BB—N2B—H2BC	122 (3)
C2A—C1A—C6A	121.8 (3)	C2B—C1B—C6B	120.7 (3)
C2A—C1A—H1A	119.1	C2B—C1B—H1B	119.6
C6A—C1A—H1A	119.1	C6B—C1B—H1B	119.6
C3A—C2A—C1A	120.0 (3)	C3B—C2B—C1B	120.0 (3)
C3A—C2A—H2A	120.0	C3B—C2B—H2B	120.0
C1A—C2A—H2A	120.0	C1B—C2B—H2B	120.0
C2A—C3A—C4A	120.5 (3)	C2B—C3B—C4B	120.7 (3)
C2A—C3A—H3A	119.7	C2B—C3B—H3B	119.6
C4A—C3A—H3A	119.7	C4B—C3B—H3B	119.6
C3A—C4A—C5A	122.6 (3)	C3B—C4B—C5B	120.4 (3)
C3A—C4A—H4A	118.7	C3B—C4B—H4B	119.8
C5A—C4A—H4A	118.7	C5B—C4B—H4B	119.8
N2A—C5A—C4A	122.8 (3)	N2B—C5B—C4B	121.2 (3)
N2A—C5A—C6A	119.4 (3)	N2B—C5B—C6B	119.6 (3)
C4A—C5A—C6A	117.6 (3)	C4B—C5B—C6B	119.1 (3)
N1A—C6A—C1A	126.1 (3)	C1B—C6B—C5B	119.1 (3)
N1A—C6A—C5A	116.4 (3)	C1B—C6B—N1B	124.2 (3)
C1A—C6A—C5A	117.4 (3)	C5B—C6B—N1B	116.6 (2)
N1A—C7A—C8A	122.3 (3)	N1B—C7B—C8B	122.5 (2)
N1A—C7A—H7A	118.9	N1B—C7B—H7B	118.8
C8A—C7A—H7A	118.9	C8B—C7B—H7B	118.8
C13A—C8A—C9A	119.1 (3)	C13B—C8B—C9B	119.1 (3)
C13A—C8A—C7A	118.7 (2)	C13B—C8B—C7B	120.7 (2)
C9A—C8A—C7A	122.3 (3)	C9B—C8B—C7B	120.2 (2)
C10A—C9A—C8A	120.4 (3)	C10B—C9B—C8B	119.9 (3)
C10A—C9A—H9A	119.8	C10B—C9B—H9B	120.1
C8A—C9A—H9A	119.8	C8B—C9B—H9B	120.1
C9A—C10A—C11A	120.9 (3)	C9B—C10B—C11B	121.0 (3)
C9A—C10A—H10A	119.5	C9B—C10B—H10B	119.5
C11A—C10A—H10A	119.5	C11B—C10B—H10B	119.5
C10A—C11A—C12A	119.9 (3)	C10B—C11B—C12B	120.2 (3)
C10A—C11A—H11A	120.0	C10B—C11B—H11B	119.9
C12A—C11A—H11A	120.0	C12B—C11B—H11B	119.9
O2A—C12A—C11A	124.9 (2)	O2B—C12B—C11B	125.0 (2)
O2A—C12A—C13A	115.9 (2)	O2B—C12B—C13B	116.1 (2)
C11A—C12A—C13A	119.2 (2)	C11B—C12B—C13B	118.9 (3)
O1A—C13A—C8A	118.5 (2)	O1B—C13B—C8B	118.1 (2)
O1A—C13A—C12A	120.9 (2)	O1B—C13B—C12B	120.9 (2)
C8A—C13A—C12A	120.5 (2)	C8B—C13B—C12B	120.8 (2)
O1A—C14A—C15A	107.6 (2)	O1B—C14B—C15B	107.4 (2)
O1A—C14A—H14A	110.2	O1B—C14B—H14C	110.2
C15A—C14A—H14A	110.2	C15B—C14B—H14C	110.2
O1A—C14A—H14B	110.2	O1B—C14B—H14D	110.2
C15A—C14A—H14B	110.2	C15B—C14B—H14D	110.2
H14A—C14A—H14B	108.5	H14C—C14B—H14D	108.5
C16A—C15A—C20A	118.7 (3)	C20B—C15B—C16B	118.9 (2)
C16A—C15A—C14A	121.1 (2)	C20B—C15B—C14B	120.1 (2)
C20A—C15A—C14A	120.1 (2)	C16B—C15B—C14B	121.0 (2)
C15A—C16A—C17A	120.4 (3)	C15B—C16B—C17B	120.1 (3)
C15A—C16A—H16A	119.8	C15B—C16B—H16B	119.9
C17A—C16A—H16A	119.8	C17B—C16B—H16B	119.9
C18A—C17A—C16A	119.9 (3)	C18B—C17B—C16B	120.1 (3)
C18A—C17A—H17A	120.1	C18B—C17B—H17B	120.0
C16A—C17A—H17A	120.1	C16B—C17B—H17B	120.0
C17A—C18A—C19A	120.4 (3)	C17B—C18B—C19B	120.2 (3)
C17A—C18A—H18A	119.8	C17B—C18B—H18B	119.9
C19A—C18A—H18A	119.8	C19B—C18B—H18B	119.9
C18A—C19A—C20A	119.5 (3)	C20B—C19B—C18B	119.7 (3)
C18A—C19A—H19A	120.2	C20B—C19B—H19B	120.2
C20A—C19A—H19A	120.2	C18B—C19B—H19B	120.2
C19A—C20A—C15A	121.0 (3)	C19B—C20B—C15B	121.1 (3)
C19A—C20A—H20A	119.5	C19B—C20B—H20B	119.5
C15A—C20A—H20A	119.5	C15B—C20B—H20B	119.5
O2A—C21A—H21A	109.5	O2B—C21B—H21D	109.5
O2A—C21A—H21B	109.5	O2B—C21B—H21E	109.5
H21A—C21A—H21B	109.5	H21D—C21B—H21E	109.5
O2A—C21A—H21C	109.5	O2B—C21B—H21F	109.5
H21A—C21A—H21C	109.5	H21D—C21B—H21F	109.5
H21B—C21A—H21C	109.5	H21E—C21B—H21F	109.5
			
C6A—C1A—C2A—C3A	−1.4 (5)	C6B—C1B—C2B—C3B	−0.3 (4)
C1A—C2A—C3A—C4A	3.1 (5)	C1B—C2B—C3B—C4B	−0.7 (4)
C2A—C3A—C4A—C5A	−1.9 (5)	C2B—C3B—C4B—C5B	0.4 (4)
C3A—C4A—C5A—N2A	−175.4 (3)	C3B—C4B—C5B—N2B	177.2 (3)
C3A—C4A—C5A—C6A	−1.0 (4)	C3B—C4B—C5B—C6B	0.8 (4)
C7A—N1A—C6A—C1A	−30.8 (4)	C2B—C1B—C6B—C5B	1.5 (4)
C7A—N1A—C6A—C5A	154.2 (3)	C2B—C1B—C6B—N1B	178.1 (3)
C2A—C1A—C6A—N1A	−176.5 (3)	N2B—C5B—C6B—C1B	−178.2 (3)
C2A—C1A—C6A—C5A	−1.6 (4)	C4B—C5B—C6B—C1B	−1.7 (4)
N2A—C5A—C6A—N1A	−7.4 (4)	N2B—C5B—C6B—N1B	4.9 (4)
C4A—C5A—C6A—N1A	178.1 (3)	C4B—C5B—C6B—N1B	−178.6 (2)
N2A—C5A—C6A—C1A	177.2 (3)	C7B—N1B—C6B—C1B	33.0 (4)
C4A—C5A—C6A—C1A	2.7 (4)	C7B—N1B—C6B—C5B	−150.3 (3)
C6A—N1A—C7A—C8A	179.2 (2)	C6B—N1B—C7B—C8B	−178.7 (2)
N1A—C7A—C8A—C13A	178.0 (3)	N1B—C7B—C8B—C13B	−179.1 (3)
N1A—C7A—C8A—C9A	−1.7 (4)	N1B—C7B—C8B—C9B	−0.5 (4)
C13A—C8A—C9A—C10A	0.8 (4)	C13B—C8B—C9B—C10B	−0.7 (4)
C7A—C8A—C9A—C10A	−179.4 (3)	C7B—C8B—C9B—C10B	−179.3 (3)
C8A—C9A—C10A—C11A	−1.3 (4)	C8B—C9B—C10B—C11B	0.8 (4)
C9A—C10A—C11A—C12A	1.0 (4)	C9B—C10B—C11B—C12B	−0.4 (4)
C21A—O2A—C12A—C11A	−3.6 (4)	C21B—O2B—C12B—C11B	−2.5 (4)
C21A—O2A—C12A—C13A	177.3 (2)	C21B—O2B—C12B—C13B	176.8 (2)
C10A—C11A—C12A—O2A	−179.3 (3)	C10B—C11B—C12B—O2B	179.1 (3)
C10A—C11A—C12A—C13A	−0.1 (4)	C10B—C11B—C12B—C13B	−0.1 (4)
C14A—O1A—C13A—C8A	119.4 (3)	C14B—O1B—C13B—C8B	−120.0 (3)
C14A—O1A—C13A—C12A	−63.8 (3)	C14B—O1B—C13B—C12B	64.1 (3)
C9A—C8A—C13A—O1A	176.9 (2)	C9B—C8B—C13B—O1B	−175.8 (2)
C7A—C8A—C13A—O1A	−2.9 (4)	C7B—C8B—C13B—O1B	2.8 (4)
C9A—C8A—C13A—C12A	0.0 (4)	C9B—C8B—C13B—C12B	0.1 (4)
C7A—C8A—C13A—C12A	−179.7 (2)	C7B—C8B—C13B—C12B	178.7 (2)
O2A—C12A—C13A—O1A	2.1 (4)	O2B—C12B—C13B—O1B	−3.2 (4)
C11A—C12A—C13A—O1A	−177.1 (2)	C11B—C12B—C13B—O1B	176.1 (2)
O2A—C12A—C13A—C8A	178.8 (2)	O2B—C12B—C13B—C8B	−179.0 (2)
C11A—C12A—C13A—C8A	−0.4 (4)	C11B—C12B—C13B—C8B	0.3 (4)
C13A—O1A—C14A—C15A	−156.0 (2)	C13B—O1B—C14B—C15B	155.0 (2)
O1A—C14A—C15A—C16A	−102.4 (3)	O1B—C14B—C15B—C20B	−65.1 (3)
O1A—C14A—C15A—C20A	76.5 (3)	O1B—C14B—C15B—C16B	113.6 (3)
C20A—C15A—C16A—C17A	0.0 (4)	C20B—C15B—C16B—C17B	−0.1 (4)
C14A—C15A—C16A—C17A	178.8 (2)	C14B—C15B—C16B—C17B	−178.8 (2)
C15A—C16A—C17A—C18A	−1.5 (4)	C15B—C16B—C17B—C18B	1.5 (4)
C16A—C17A—C18A—C19A	1.4 (4)	C16B—C17B—C18B—C19B	−1.7 (4)
C17A—C18A—C19A—C20A	0.1 (4)	C17B—C18B—C19B—C20B	0.4 (4)
C18A—C19A—C20A—C15A	−1.7 (4)	C18B—C19B—C20B—C15B	1.0 (4)
C16A—C15A—C20A—C19A	1.6 (4)	C16B—C15B—C20B—C19B	−1.2 (4)
C14A—C15A—C20A—C19A	−177.3 (2)	C14B—C15B—C20B—C19B	177.5 (2)

### Hydrogen-bond geometry (Å, °)

**Table:**

*D*—H···*A*	*D*—H	H···*A*	*D*···*A*	*D*—H···*A*
N2A—H2AC···N1A	0.92 (3)	2.31 (3)	2.735 (4)	108 (2)
N2A—H2AC···N2B^i^	0.92 (3)	2.49 (3)	3.229 (4)	138 (3)
N2B—H2BC···N1B	0.90 (2)	2.29 (3)	2.726 (3)	109 (2)
C7A—H7A···O1A	0.95	2.43	2.765 (3)	101
C7B—H7B···O1B	0.95	2.46	2.790 (3)	100
C14A—H14A···O2A	0.99	2.43	2.895 (3)	108
C14B—H14D···O2B	0.99	2.45	2.903 (3)	107
C21A—H21C···Cg1^ii^	0.98	2.96	3.511 (3)	117
C21B—H21F···Cg2^iii^	0.98	2.81	3.739 (3)	159
C10A—H10A···Cg3^ii^	0.95	2.60	3.500 (3)	159
C21B—H21E···Cg4^iv^	0.98	2.80	3.433 (3)	123
C21A—H21B···Cg5^v^	0.98	2.96	3.844 (4)	150
C10B—H10B···Cg6^iv^	0.95	2.66	3.587 (3)	165
N2B—H2BC···Cg6^vi^	0.90 (2)	2.83 (3)	3.288 (3)	113 (2)

Symmetry codes: (i) *x*, −*y*+3/2, *z*−1/2; (ii) −*x*+2, *y*−1/2, −*z*+1/2; (iii) *x*−1, *y*, *z*; (iv) −*x*+1, *y*+1/2, −*z*+1/2; (v) *x*+1, *y*, *z*; (vi) *x*, −*y*+1/2, *z*−1/2.
